# They are not all the same: Determinants of attendance across different sectors in a stadium

**DOI:** 10.1371/journal.pone.0289331

**Published:** 2023-07-27

**Authors:** Angel Barajas, Thadeu Gasparetto

**Affiliations:** 1 Facultad de Ciencias Empresariales y Turismo, Universidad de Vigo, Ourense, Spain; 2 Carnegie School of Sport, Leeds Beckett University, Leeds, United Kindgom; Privatuniversität Schloss Seeburg: Privatuniversitat Schloss Seeburg, AUSTRIA

## Abstract

Previous research inspecting the demand for tickets for professional sports has mostly used aggregate data in their estimations. In a nutshell, it implies that all fans would be driven by the same determinants. In this research, we test whether this hypothesis holds. We analyse all first-tier Brazilian League home matches of both *Flamengo* and *Fluminense* at the iconic stadium *Maracanã* (Brazil) from 2014 to 2019. Ordinary Least Square regressions model individual equations for each sector for comparing their determinants. Our empirical results offer evidence that ticket price and Uncertainty of Outcome have different impact on demand for tickets according to the sector. Further research is encouraged to inspect whether similar behavior is detected in other settings.

## 1. Introduction

A football fan has habitually many options when purchasing a ticket for attending a match. Different sectors, seats, fixtures, and hospitality packages–among other factors–may influence the consumption process of a football event. However, most of the previous research inspected the demand for tickets using aggregated data [1], which would imply a common behavioral intention among all fans across the stadium.

One of the biggest methodological challenges that researchers face when modelling stadium attendance is the lack of disaggregated data. Although the concept of aggregated data is somewhat straightforward, the term ‘disaggregated data’ may refer to several dimensions, at least for demand for tickets, and its definition is rather vague. First, data can be disaggregated across different decisions, such as the initial purchase decision and subsequent admission or no-show decisions. This allows for a more nuanced analysis of the factors influencing each stage of the process. Second, disaggregation can occur at various population levels, including the micro/individual, meso, and macro levels. By examining data at these different levels, we can gain insights into individual behavior, group dynamics, and broader contextual factors. Third, disaggregation across time, such as match-by-match or seasonal analysis, provides an understanding of temporal dynamics and allows for the identification of trends and patterns. Finally, data can be disaggregated at the spatial level. It considers different sectors within a stadium and enables a detailed analysis of fan preferences, behaviors, and demand patterns, providing valuable insights into the dynamics of attendance and engagement within specific areas of the venue.

There are, indeed, a few attempts trying to deal with potential differences between groups of fans. Some works, for instance, exclude the season ticket holders from their equations [2–4], while others try to predict the season ticket holders’ behavior itself [5–9]. On the other hand, some research pursued to inspect the differences among groups of fans: there is evidence that TV broadcast would reduce the ticket demand for pay-at-the-gate home supporters, but it would not affect season ticket holders [10], as well as other that indicates differences in the demand for standings and seated attendance–where the former is attracted by the significance of the match and the current performance of the team, while the latter is committed with the club’s historical record [11]. Differences in preferences between British home and away fans are also found recently. For instance, the magnitude of the impact of public holidays, fixtures and competitive intensity is diverse for each group [12]. Furthermore, data on no-show behaviour could also be considered as a form of disaggregated data and some latest research has been examining its impact on stadium attendance [7, 8, 13, 14].

The research concerning behaviour of sports spectators considering prices within a single stadium is also very scarce. There is a single evidence in baseball where fans can value seats differently even if the only difference is the side of the seat in the stadium [15]. In other industries like theater, opera, or ballet, there are some studies about the demand for different seat categories–locations at the theater. Schimmelpfennig [16] concluded that there exist opportunities in some locations for price cuts for increased both attendance and revenue. That was the case for the rear part of the Amphitheatre in which it could be possible to increase -differently- the median attendance for two different ballet performances [16] In the theatrical industry, demand elasticities vary within seating categories [17]. The distance from the front in theaters plays a crucial role in identifying the optimal number of high-price seating rows. Distance significantly decreases demand and there are fewer incentives in paying higher prices for seats further in the back. Moreover different types of performances have different distance sensitivities [18]. Somehow, ticket pricing and demand may have commonalities between sports and other entertainment industries. Still, it is worth remarking that price elasticity may vary across the different categories of seats.

In this research, we delve into the disaggregated data at a spatial level within the stadium. In other words, we investigate the ticket demand by sectors, aiming to explore variations in fan preferences and behaviors based on their seating locations. This approach allows for a more detailed understanding of how different sections of the stadium contribute to overall ticket sales and fan engagement. To the best of our knowledge, there are only few works on football that previously addressed similar issue. A manuscript investigating Preston North End matches over two seasons (1955/56 and 1991/92) [19], a discussion paper which inspects the differences in the determinants of home and away fans using extensive data in England–mostly from lower divisions [12], a study that focused on the flow of away fans for football matches in Spain [20], and a recent paper which inspects the differences in preferences among daily ticket purchasers and season ticket holders [21].

However, we were able to gather more detailed and granular data from Brazilian football. In fact, we could identify the total number of fans who purchased tickets for each section of the stadium. This made it possible to compare the determinants between different groups of local fans based on their location within the stadium, not only between home and away fans or daily purchasers or season ticket holders, which had already been by previous authors.

Therefore, the current research aims to shed light on whether the determinants of stadium demand vary according to the sector of the purchased ticket. We believe that if differences are observed across sectors–suggesting that fans are driven by distinct determinants or, in other words, that factors impact differently a group of fans in a sector more than in another–it would lead to a new avenue for further research studying this subject. At the same time, this paper offers a novel contribution to the knowledge on demand for tickets in Brazil, following the previous works which evidenced and discussed the low attendance rates at football matches in that country [22–25]. We assume that understanding the breakdown of sections within football stadiums might help clubs to optimize their ticket sales and, hence, increase the demand for tickets and match day revenue.

The paper is structured as follows: The Methods section provides a detailed description of the setting and introduces the modelling approach and the variables used in the analysis. The Results and Discussion section presents and discusses our empirical findings, which shed light on the differences that emerged. The conclusion draws final remarks on the study and suggests potential avenues for future research. Our paper contributes to the existing literature by providing new empirical evidence and insights on the potential differences in the demand for tickets using disaggregated data. Overall, this study offers a valuable contribution to the field and opens up new research opportunities.

## 2. Methods

### 2.1. Setting

We focus our study on the first tier of the Brazilian League due to the availability of data. That tournament is played in a double-round robin design as most European domestic competitions. For our research, we inspected the home matches played in a single stadium, *Maracanã*, the largest and most iconic in that country. This choice–for a single stadium–is made for a homogeneous sample selection. In fact, one cannot compare the behavioral intentions of fans when stadium characteristics (and stadiums themselves) are different. We inspected the home matches of *Flamengo* and *Fluminense* from 2014 to 2019.

The present capacity of *Maracanã* is 78.838 attendees, a quantity far away from the official record– 183,341 tickets sold for Brazil x Paraguai in 1969 –and the unofficial estimation of attendees–about 200,000 supporters in the FIFA World Cup Final in 1950. The stadium is currently divided into four main sectors: *North*, *South*, *East*, and *West*. The *North* and *South* sectors are divided into three levels (Superior I and II, and Inferior), while *East* and *West* are divided into two levels (Superior and Inferior). The levels take into account the distance between seats and the playing field, where the Inferior is the closest to the pitch. Additionally, the stadium has a small VIP sector called *Maracanã+*–located within the *West* sector–and the so-called *Cadeiras Cativas* (Captive Seats). The attendance of Captive Seats is not studied in this work since they do not face similar demand as other sectors. Captive Seats have *owners*, who have the right to attend any match played at Maracanã regardless of the teams competing. The stadium map can be seen on the Maracanã official webpage (https://www.estadiodomaracana.com.br/setores-do-estadio-do-maracana/).

Unfortunately, there is no official information regarding the maximum capacity of each sector (e.g., stadium’s website, clubs’ website, Rio de Janeiro City Council website, etc.). However, Brazilian sports newspapers often announce that the two largest sectors (North and South) have 22,000 seats each–but the capacity of the East and West sectors has not been found.

The most engaged supporters of Flamengo are habitually located in the *North* sector, while alike Fluminense fans are regularly located in the *South* sector. Hence, when Flamengo is the home team, the away fans are located in the *South* sector, whilst when Fluminense is the home team, the away fans are located in the *North* sector. In a match between these two clubs (regardless of who is the home club in the fixture) fans of Flamengo stay in the *North* and Fluminense supporters in the *South*, while sectors *West* and *East* are mixed or exclusive for one club or another, according to the competition rules or an agreement between these clubs. We have called these sectors–*North* for Flamengo and *South* for Fluminense–*Ultras*. The most engaged supporters in Brazil are called *torcidas organizadas* and are like ultras in Europe.

### 2.2. Model and variables

The econometric approach consists of Ordinary Least Square (OLS) regressions with home team and season fixed effects. The dependent variable is the logarithm of the attendance *a* on each sector *s* for each club *i* on season *t*. The regression models one individual equation for each sector for comparing their determinants. The variables of interest are: the logarithm of the ticket price (*p*), and the Uncertainty of Outcome (*UO*) matrix, which includes the home win probability (*hwp*) in the first model and adds its squared term (*hwp*^*2*^) in the second one. Control variables (*CV*) include the weekdays (*w*) and a specific dummy for each away team (*j*). The general model is following:

$$a_{sit}=\beta_{0}+\beta_{1}p_{sit}+\delta UO_{sit}+\varpi CV_{sit}+\varepsilon$$

The ticket price (*p*) is taken in logarithm–similar to the dependent variable–, which allows us to interpret the elasticity. The coefficient of price (*p*) is expected to be significant and negative, following the law of supply and demand. However, our hypothesis is that the magnitude of the impact as well as the elasticity might differ across sectors, which would constitute one of the main contributions of our research.

Nonetheless, the expected impact of the uncertainty of outcome is unclear. Although the classic Uncertainty of Outcome Hypothesis suggests that fans are attracted by higher levels of uncertainty [26], a recent meta-analysis indicates that there is no consensus in this regard [27]. For this reason, we estimated both linear and quadratic forms in our estimations. If only linear impact is identified, it would indicate that the demand for tickets significantly increases when the home team has a larger probability of winning. On the other hand, if a quadratic effect is identified, the probability of winning would have a positive (negative) impact on the demand until a certain point and then decrease (increase) after this tipping point. Likewise the price, the UO may drive demand differently according to the sector.

However, it is important to emphasise that the interpretation of the coefficients depends on the sector under analysis due to the nature of the fans–highly engaged (*Ultras*), casuals (*East/West*) or away (*Away*). For instance, a significant positive impact of UO in the *Ultras* or *East/West* sectors implies that local fans are attracted by higher chances of their team winning, but the same positive impact of UO in the *Away* sector implies that away fans tend to significantly attend more matches when they are less likely to win (underdogs).

The dependent variable (*a*) includes the logarithm of all tickets sold for a sector (*s*) regardless of the amount purchased on each level (i.e., superior/inferior). There are four main reasons for this choice: (1) Clubs often charge identical prices for superior/inferior levels within a same sector; (2) for the particular cases when some levels are more expensive/cheaper than others, this variation will be absorbed by the variable price (*p*) in the equation; (3) no single sector or level reached its (*hypothetical*) full capacity over the whole sample; and (4) fans do not choose their seats during the purchase process, they choose the sector instead–indeed, they will stay wherever they want within the sector chosen regardless the seat displayed in the ticket.

We recognize that demand-based research on European football usually eliminates the season ticket holders from their equations, but we could not do it in our study. Season tickets have not been a popular ticketing practice in Brazilian football–although it is becoming more popular in recent times. In fact, most football clubs in Brazil are considered as non-profitable associations, where their presidents are chosen via elections by the *socios* (club members)–a similar structure observed in a few European clubs, such as Real Madrid or Barcelona. *Socios* are supporters that pay monthly/yearly membership and have the right to vote. However, differently from a season ticket, the *socio membership* does not guarantee tickets to football matches. Some do have specific discounts when purchasing tickets, but it is clearly different from what commonly happens in European football. Since we are not able to identify the *socios* as well as they still have to pay for a match they are willing to attend, we assume that their inclusion does not constitute any issue.

Taking into account that none of the sectors reached its full capacity, we assume that we are likely capturing the first decision of fans–those who directly bought the tickets from the clubs. We acknowledge that no-show behaviour is increasing worldwide, as well as the existence of secondary markets for football tickets, but the lack of information in these regards does not allow us to address these elements here. Moreover, a recent paper analyzing football in Brazil has offered evidence that price bundling strategies have been implemented by a few clubs and this, indeed, statistically impact the consumption of football tickets [28]. Nonetheless, in our dataset such phenomenon was not possible to identify and, therefore, we acknowledge that as a current limitation. However, we believe that it does not represent a big issue since it tends to correspond to only a small number of cases for both clubs under research.

In regards to the econometric exercise, the OLS models are chosen due to the characteristics of the data. We acknowledge that some recent papers have implemented censored regression models (e.g., Tobit) in their research [29–31]. This is an appropriate choice when the dependent variable is censored above or below a certain threshold–in sports research, the threshold use to be when demand reaches the maximum capacity or particularly high levels, like 95% of the total. Nonetheless, none of the matches or individual sectors have faced capacity constraints during our research period. In other words, both Flamengo and Fluminense did not experience any sold-out tickets in their Brazilian League matches from 2014 to 2019. Therefore, a censored model is not the most suitable tool and OLS regressions have been chosen.

A total of ten OLS models are carried out: 1 and 2 examine the demand for *Ultras* sector, 3 and 4 for *Away* sector, 5 and 6 to *East* sector, 7 and 8 to *West* sector and 9 and 10 to *Maracanã+*. As explained above, the first model for each sector considers a linear relationship between UO and demand, while the second model explores whether there is a quadratic relationship between these variables.

## 3. Results and discussion

**Fig 1:**
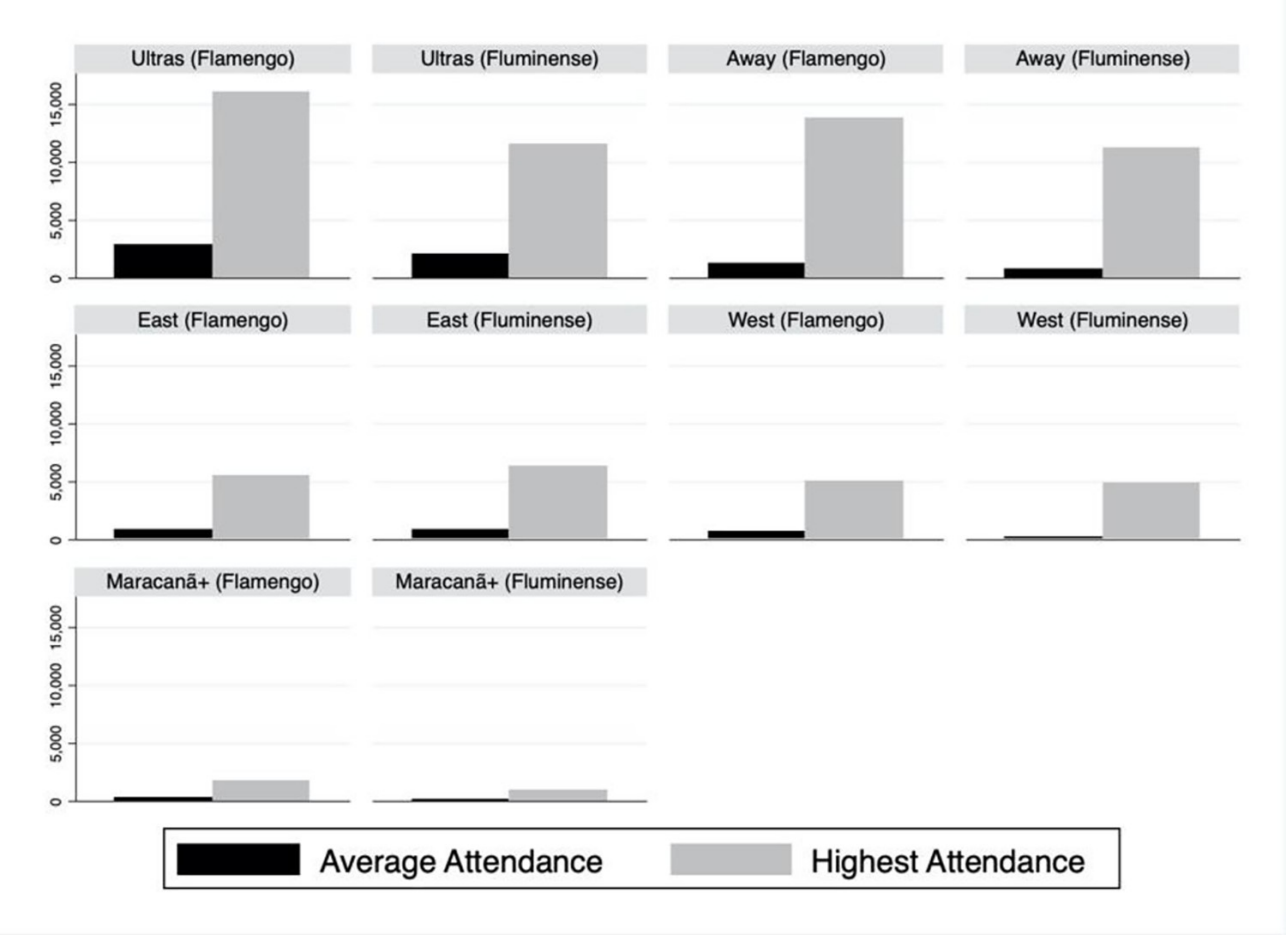
Average and highest attendance by clubs and sectors.

Fig 1 exhibits both average and the highest attendance by clubs in each sector. As one can see, Flamengo experiences slightly larger attendance levels than Fluminense for almost all sectors–the exception is in the *East*–but it does not translate into significant differences between them, except for the *Maracanã+* where Flamengo fans show significantly greater attendance levels.

Table 1 shows the descriptive statistics by clubs and sectors. The attendance figures demonstrate that censored models were not needed. The largest observation represents 16,024 attendees in the *North* sector from Flamengo supporters. Taking into account that the maximum capacity in that sector is about 22,000 seats, it falls short of its full capacity. The same is observed in all the other sectors, which confirms that capacity constraint is not an issue for both Flamengo and Fluminense. There were some cases when the minimum attendance displays zero attendees. Unfortunately, we were not able to identify whether the clubs decided to close the sector or no attendees purchased tickets for these sectors. Nonetheless, regardless of the reason, we assume that it does not constitute an issue as these observations dropped after the log transformation. The remaining zeros represent the lower bound of the data (in logarithm), not an absence of fans.

**Table 1:** Descriptive statistics.

	*Flamengo*						*Fluminense*				
Variable	Obs	Mean	Std Dev	Min	Max		Obs	Mean	Std Dev	Min	Max
						***Ultras***					
**Attendance**	411	2902.09	3316.92	0	16024		284	2134.10	2076.11	0	11608
**Att. (log)**	398	6.85	2.14	0	9.68		276	6.91	1.88	0	9.36
**Price**	411	32.96	18.81	5	120		284	26.41	14.55	0	60
**Price (log)**	411	3.35	.54	1.61	4.79		281	3.12	.62	.92	4.09
**H. Win P.**	411	.56	.12	.33	.93		284	.50	.12	.21	.76
						***Away***					
**Attendance**	443	1335.42	2168.14	0	13732		234	769.28	1533.25	0	11206
**Att. (log)**	397	5.92	2.08	0	9.53		193	5.67	1.69	.69	9.32
**Price**	443	37.16	24.66	0	140		234	27.34	16.39	0	80
**Price (log)**	405	3.55	.56	1.61	4.94		217	3.23	.59	.92	4.38
**H. Win P.**	443	.59	.12	.33	.93		234	.51	.12	.21	.76
						***East***					
**Attendance**	752	922.41	1043.66	0	5450		478	818.74	1010.02	0	6252
**Att. (log)**	720	5.79	1.93	0	8.60		390	6.02	1.79	0	8.74
**Price**	752	58.22	45.92	0	335		478	23.44	19.59	0	90
**Price (log)**	743	3.86	.64	1.61	5.81		413	3.05	.75	.92	4.50
**H. Win P.**	752	.62	.12	.33	.93		478	.49	.12	.21	.76
						***West***					
**Attendance**	216	686.18	958.82	0	4977		54	276.11	843.34	0	4908
**Att. (log)**	155	5.90	1.90	.69	8.51		13	5.74	2.21	2.30	8.50
**Price**	216	52.47	38.65	0	165		54	12.78	24.37	0	80
**Price (log)**	186	3.95	.60	1.61	5.11		15	3.65	.68	2.30	4.38
**H. Win P.**	216	.62	.12	.33	.93		54	.48	.13	.21	.65
						***Maracanã+***					
**Attendance**	291	277.26	347.45	0	1777		122	136.44	162.95	0	1015
**Att. (log)**	272	4.86	1.51	0	7.48		107	4.56	1.08	1.61	6.92
**Price**	291	135.02	102.19	0	635		122	69.06	39.13	0	160
**Price (log)**	288	4.68	.70	1.61	6.45		107	4.29	.39	3.81	5.07
**H. Win P.**	291	.62	.12	.33	.93		122	.52	.13	.21	.76

Regarding the ticket prices, one may note the presence of free tickets–with price equal zero. Similarly to the attendance, these observations were excluded after the log transformation. Overall, one can see that Flamengo sets higher ticket prices than Fluminense–both the average and maximum are higher for all sectors. As expected for a VIP area, the *Maracanã+* has the highest average ticket price among all sectors. Two curious behaviours in pricing strategy are observed between the clubs: Flamengo charges the cheapest average ticket for its own supporters in the *Ultras* sector, while this is the most expensive one (on average) for Fluminense fans. However, Fluminense charges, on average, higher ticket prices for away fans than for its own supporters, but Flamengo does not do the same.

Table 2 displays the outputs of regression models. The results show evidence that the explanatory factors impact differently the ticket consumption according to the sector. Tickets price, for instance, is negatively associated with lower demand for tickets in the sectors *Ultras* and *East*, while in others it seems to be irrelevant. Our empirical results show evidence that the demand for tickets is inelastic for both *Ultras* and *East* sectors, but the magnitude varies between them. The most engaged fans (commonly seated in the *Ultras* sector) are more sensitive to prices: an increase of 1% implies a 0.89% reduction in the demand, *ceteris paribus*. On the other hand, fans located in the *East* sector are less sensitive to a price increase, once 1% of the increase in prices produces about a 0.38% reduction in the demand for tickets, holding all constant.

**Table 2:** Regression outputs of stadium demand models by sectors.

VARIABLES	Ultras	Ultras	Away	Away	East	East	West	West	Maracana+	Maracana+
Price (log)	-0.889***	-0.890***	0.0522	0.0495	-0.376***	-0.381***	-0.107	-0.107	-0.0843	-0.0904
	(0.148)	(0.148)	(0.152)	(0.151)	(0.0922)	(0.0922)	(0.354)	(0.355)	(0.143)	(0.143)
H Win Prob	2.454**	-0.381	3.573***	-12.11**	1.693**	-4.122	5.795*	5.780	0.413	-4.114
	(1.046)	(5.183)	(1.133)	(5.703)	(0.826)	(3.846)	(2.958)	(19.48)	(0.964)	(5.087)
H Win Prob		2.673		14.93***		5.429		0.0158		4.261
		(4.786)		(5.323)		(3.507)		(20.61)		(4.702)
i.Fluminense	0.0198	0.0402	0.223	0.329	0.00851	0.0585	-1.214	-1.214	-0.628***	-0.596***
	(0.218)	(0.221)	(0.217)	(0.219)	(0.181)	(0.183)	(1.330)	(1.382)	(0.226)	(0.229)
Weekday	Yes	Yes	Yes	Yes	Yes	Yes	Yes	Yes	Yes	Yes
Away Club	Yes	Yes	Yes	Yes	Yes	Yes	Yes	Yes	Yes	Yes
Season FE	Yes	Yes	Yes	Yes	Yes	Yes	Yes	Yes	Yes	Yes
Constant	9.521***	10.26***	2.126*	6.174***	7.511***	9.028***	5.087	5.091	5.167***	6.565***
	(1.317)	(1.867)	(1.290)	(1.930)	(1.142)	(1.504)	(3.453)	(5.565)	(1.612)	(2.231)
Observations	674	674	590	590	1,110	1,110	168	168	379	379
R^2^	0.140	0.141	0.208	0.219	0.072	0.074	0.197	0.197	0.221	0.223
Adjusted R^2^	0.083	0.082	0.147	0.158	0.035	0.037	-0.009	-0.016	0.132	0.131

Standard errors in parentheses

*** p<0.01

** p<0.05

* p<0.1

These empirical results suggest a practical implication for decision markers of Flamengo and Fluminense. The descriptive statistics indicate several empty seats in all their matches. Considering that fans are not very sensitive to changes in ticket prices, both clubs could set higher ticket prices and would likely experience similar attendance levels. This, in theory, would lead to larger match-day revenues.

The outputs also indicate important findings regarding the impact of the uncertainty of outcome. The first equation suggests that in the sectors *Ultras*, *Away* and *West* supporters are highly interested in the home win probability–higher attendance as much favorite the home team is. It could be understood that local fans (located in *Ultras* and *West*) are significantly attracted by higher winning probabilities–in other words, more local fans tend to attend as more favourite the home club is.

Additionally, this particular result would indicate that the demand for tickets by away fans would be greater as lower their likelihood of winning. It could be assumed that away fans are interested in attending matches when they are clear underdogs. Nonetheless, the second equation reveals a significant quadratic relationship between home win probability and demand for tickets in the *Away* sector. This result suggests that away fans are not only interested in attending when they are underdogs, but also when they are the favourites in the match. Based on the regression output, the tipping point is when the home team has a probability to win of 40.55%. Therefore, very competitive matches would not attract many away supporters. Previous research indicates differences between the demand for tickets by home and away fans regarding the position of their teams in the league table and their current performances [12], but nothing significant has been observed in terms of differences in winning probabilities. In this sense, we assume that our research offers novel findings in this regard.

There is no clear consensus in the literature regarding the impact of UO on attendance [27]. Indeed, there are empirical evidence that UO impacts positively and negatively the ticket consumption. Recently, most of the empirical papers has been addressing this relationship from the reference-dependent preferences perspective [32], and the empirical findings suggest an increase in overall demand by high and low home win probability [33–35]. Nonetheless, once nearly all papers work with aggregated-level data, we advise that some of the overall results might be biased. Our empirical findings suggest differences between highly engaged, casual and away fans in Brazilian football and we recommend a careful interpretation of the previous empirical results for policy implementations once some of them could mislead crucial decisions. For instance, the price elasticity might be specific for each price category, while the uncertainty of outcome shall impact differently the fans according to their level of engagement. Lastly, the previous findings suggesting the significant quadratic relationship between winning probability and overall demand could be re-examined, once based on our findings it may not be constant for all fans: indeed, the left portion of the demand curve might be driven by the away fans or by only one group of local supporters that do show loss aversion behaviour.

The fact that both price and uncertainty of outcome do not impact ticket consumption in the VIP sector raises an interesting enquiry: What are the determinants of attendance for VIP tickets? Unfortunately, our research cannot answer this question, although our preliminary findings show evidence that price and uncertainty of outcome do not impact. However, further research is needed to shed light on this matter. At the same time, the free tickets were excluded from our equations, and future investigations could try to understand the key factors for this unique demand that has been overlooked until the date.

We acknowledge that the present research has certain limitations. We do consider that the panel data analysed here is large enough for the econometric exercise performed, but further research could employ a larger sample size, which would allow the addition of a higher number of explanatory variables. Since we examined only two football clubs, we could not include some match characteristics (e.g., current performance, derby, etc.), as it would raise multicollinearity issues with other variables (i.e., winning probabilities, home team dummies and away teams fixed effects). Moreover, we recognize that some missing factors tend to influence the demand for tickets (e.g., substitutes, weather conditions, etc.), but, unfortunately, we were not able to gather such information. However, we do not consider omitted-variable bias as an issue of our modelling. We do believe that the inclusion of these factors would, indeed, increase the explanatory power of the models, but would not change the significant effects observed in the other independent variables.

Our research, by no means, tries to end the discussion about determinants of attendance for different sectors. Instead, our goal is to encourage further research by adding new sportive- and economic-specific variables, much larger datasets, and studies examining whether similar behavior is found in different settings, such as North American Major Leagues, European football, and different sports disciplines. Moreover, while our study acknowledges the importance of disaggregated data, we recognize that our data are not fully disaggregated, as some aspects remain aggregated across certain decisions. However, it is essential to recognise the value of the disaggregated data we have utilized and the insights it provides while acknowledging the potential for further disaggregation in future research. Nonetheless, we show support for the statement from previous literature reviews about the need for more demand research on overlooked football leagues [1, 36]. We can luckily find few attempts to model attendance demand on South American [22–25, 37], African [38, 39] or Asian [21, 40–42] football competitions, but more can be done and the literature would certainly benefit from the potential findings.

## 4. Conclusions

This research shows evidence that the determinants of stadium attendance are not identical across all sectors. Our empirical results suggest that factors like price and the level of uncertainty of outcome impact differently in ticket consumption for diverse groups of fans. This result contributes to the literature by adding a novel approach for inspecting ticket demand using disaggregated data. Further research shall examine larger datasets as well as include other sportive and economic elements that may impact the demand for tickets. Furthermore, a practical contribution also emerges once the findings may advise clubs to look at the specific determinants by each sector to maximise ticket consumption and, hence, revenues.

## Supporting information

S1 Data (CSV)
